# Accelerated Magnetic Seizure Therapy for Non‐Suicidal Self‐Injury in Adolescents With Bipolar Depression

**DOI:** 10.1002/cns.70880

**Published:** 2026-04-21

**Authors:** Ruiqiu Zhu, Peiyu Cao, Yilin Tang, Yucheng Lin, Yifan Wang, Yantong Li, Ran Tao, Zhuma Jin, Yuxiu Sui

**Affiliations:** ^1^ Department of Psychiatry Nanjing Brain Hospital, The Affiliated Brain Hospital of Nanjing Medical University Nanjing Jiangsu China

**Keywords:** accelerated magnetic seizure therapy, adolescents, bipolar depression, non‐suicidal self‐injury

## Abstract

**Background:**

Adolescents with bipolar depression are typically characterized by a high prevalence of non‐suicidal self‐injury (NSSI), yet effective therapies remain limited. Accelerated magnetic seizure therapy (aMST) has emerged as a promising neuromodulation technique with potential cognitive advantages. However, evidence regarding its clinical efficacy, cognitive impact, neurostructural and functional alterations in adolescents remains unclear.

**Methods:**

Adolescents diagnosed with bipolar depression and comorbid NSSI received once‐daily accelerated MST sessions. Clinical symptoms, including depression, anxiety, impulsivity, and emotion regulation, were assessed before and after aMST. Cognitive function was evaluated using the MATRICS Consensus Cognitive Battery (MCCB) and autobiographical memory test (AMT). Structural MRI and diffusion tensor imaging (DTI) were collected to compare the potential neurostructural and functional change induced by aMST.

**Results:**

Thirty‐two adolescents completed the whole aMST protocol. Following aMST, significant reductions were observed in depressive and anxiety symptoms. Improvements were also found in impulsivity and emotion regulation. Assessment of cognitive function revealed no global impairment. Significant enhancements were found in processing speed, working memory, and visual learning, while verbal learning remained largely unchanged. Autobiographical memory (AM) performance was generally preserved, with a reduction in overgeneral autobiographical memory (OGM) for positive cues. Neuroimaging analyses demonstrated no significant changes in total hippocampal volume or hippocampal subfield volumes. DTI analyses suggested increased fractional anisotropy (FA) in the right uncinate fasciculus and increased mean diffusivity (MD) in the left superior longitudinal fasciculus following aMST.

**Conclusions:**

These findings provide preliminary evidence that aMST is associated with clinical improvement and favorable cognitive function in adolescents with comorbid bipolar depression and NSSI. The stably maintained volumes of hippocampal subfield, alongside selective white matter microstructural alterations, suggest that aMST may exert targeted neuroplastic effects while preserving structures which are critical for cognition.

**Trial Registration:**

Chinese Clinical Trial Registry: ChiCTR2500114164

## Graphical abstract

Accelerated Magnetic Seizure Therapy (aMST) is clinically effective and neurocognitively safe for adolescents with comorbid Bipolar Depression and Non‐suicidal Self‐Injury. Selective white matter microstructural alterations suggest that aMST may exert targeted neuroplastic effects while preserving structures which are critical for cognition.

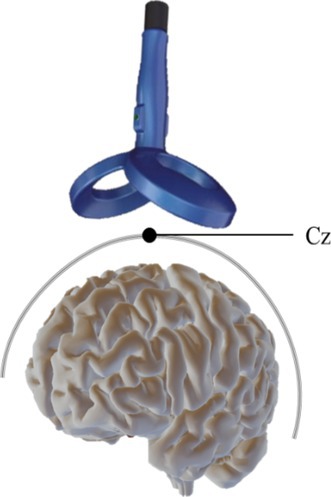

## 1 Introduction

Non‐suicidal self‐injury (NSSI) is a pattern of repeated, deliberate, and direct physical harm inflicted upon oneself without suicidal intent, which is not socially acceptable or recognized [1, 2]. NSSI is particularly prevalent among adolescents [3] and is often comorbid with several psychiatric disorders such as bipolar depression. Approximately 78.3% of adolescents with bipolar disorder engage in NSSI at least once, among which adolescents with depressive episodes show the highest rate [4, 5]. In addition, NSSI is one of the risk factors for suicidal ideation and behavior [6, 7]. The high vulnerability of adolescents with comorbid bipolar depression and NSSI represents a significant clinical challenge, particularly due to the limitation of effective treatments available to this group.

As a critical period for neurodevelopment, adolescence is highly prone to various behavioral problems and mental disorders, which may contribute to the high prevalence of NSSI during this stage of development. In fact, previous studies have demonstrated that adolescents with a history of NSSI exhibit region‐specific abnormalities in the functional connectivity among multiple brain regions involved in self‐processing, social reward processing, emotion processing, and pain processing [8]. In addition, during this period, hippocampal neurogenesis and synaptic plasticity are elevated compared with adulthood, making the hippocampus particularly susceptible to environmental stressors [9]. Longitudinal imaging studies have documented age‐related alterations in hippocampal shape and microstructure throughout childhood and adolescence, reflecting protracted maturation of this structure [10]. Importantly, disrupted hippocampal development has been linked to enduring reductions in hippocampal volume following early adversity, which may be associated with the pathophysiology of mood and anxiety disorders emerging during adolescence [11].

Current treatment strategies for bipolar depression and NSSI in adolescents mainly include pharmacotherapy and psychotherapy [12]. However, a substantial proportion of patients fail to achieve adequate symptom remission, with the depressive symptoms and self‐injurious behaviors persisting [13]. Moreover, pharmacological management of bipolar depression in adolescents is often complicated by treatment‐related adverse effects, such as metabolic shifts and the risk of treatment‐induced affective switch, which causes challenges for achieving rapid and sustained clinical improvement [14]. Electroconvulsive therapy (ECT) remains the most effective intervention for severe and treatment‐resistant mood disorders, yet its application in adolescents is constrained by concerns over cognitive adverse effects, particularly memory impairment [15, 16]. Consequently, there is an urgent clinical need to identify a novel neuromodulation technique that can reach clinical remission from adverse cognitive outcomes among adolescents with bipolar depression comorbid with a history of NSSI.

Magnetic seizure therapy (MST) is a non‐invasive convulsive neurostimulation therapy, which produces rapidly alternating strong magnetic fields to induce seizures [17]. It has been demonstrated that MST is an effective treatment for patients with bipolar depression [18]. Studies on adolescents with depression have indicated that MST is effective in achieving remission and reducing suicidal ideation while offering the additional benefit of preserving cognitive function [19, 20, 21]. Based on previous studies confirming the safety and cognitive protective effects of MST, one research has explored the efficacy of daily consecutive MST sessions which were defined as accelerated magnetic seizure therapy (aMST) and the potential cognitive damage [22]. The results further supported the feasibility and tolerability of aMST in alleviating clinical symptoms. Taken together, the clinical application of aMST may provide a new strategy toward the management of high‐risk adolescent populations, particularly those presenting with comorbid bipolar depression and NSSI.

Adolescents with bipolar depression are notably predisposed to impulsive behaviors, which substantially elevates their risk of NSSI. Currently, there are limited available interventions which can effectively control NSSI. MST has emerged as a promising convulsive neuromodulation approach, with accumulating evidence supporting its efficacy in the treatment of bipolar depression. Importantly, compared with conventional treatment modalities, aMST has been suggested to offer safer and more rapid therapeutic effects. However, evidence regarding the clinical and cognitive impact of aMST in adolescents with bipolar depression and comorbid NSSI remains unclear. In this study, we applied aMST to adolescents diagnosed with bipolar depression who had a history of NSSI. The aim of the present study was to evaluate the extent of clinical symptom remission, changes in cognitive function, and to further explore potential underlying neurobiological mechanisms through multiple neuroimaging data.

## 2 Materials and Methods

### 2.1 Participants

The experimental protocol for this clinical trial was approved by the Ethics Committee of the Affiliated Brain Hospital of Nanjing Medical University and registered on the Chinese Clinical Trial Registry site (http://www.chictr.org, number ChiCTR2500114164). All participants and their legal guardians provided written informed consent.

Assessments of prior and current self‐harming behaviors among adolescents were conducted using the Ottawa Self‐Injury Inventory (OSI) [23]. NSSI was determined based on the diagnostic criteria of the *International Classification of Diseases, 11th Revision* (ICD‐11). Inclusion criteria were: (1) aged between 14 and 18 years, (2) diagnosed with bipolar disorder, first episode, currently in a depressive episode according to the ICD‐11, (3) 17‐item Hamilton Depression Rating Scale (HDRS‐17) score of 17 or higher [24], (4) diagnosed as NSSI according to ICD‐11, (5) right‐handed, (6) able to provide informed consent. Exclusion criteria included: (1) a history of severe physical diseases (e.g., stroke, heart failure, liver failure, cancer), (2) a history of alcohol, narcotic, or other psychoactive substance use, (3) had unremovable metal implants and (4) other conditions inappropriate for participation. A total of thirty‐seven patients were assessed for eligibility, and five patients did not complete the full course of aMST due to personal or family‐related reasons.

### 2.2 Clinical Assessments

Two qualified psychiatrists assessed the clinical outcomes. Clinical assessments included HDRS‐17 [24], Hamilton Anxiety Rating Scale (HARS) [25], Chinese Version of the Difficulties in Emotion Regulation Scale‐Brief Version (DERS‐16‐CV) [26, 27] and the Chinese version of the Barratt Impulsiveness Scale (BIS‐11‐CV) [28]. The DERS‐16‐CV contained 16 self‐reported items measuring the deficits in emotional regulation. Responses to each item were designed on a five‐point Likert scale from 1 (“almost never”) to 5 (“almost always”). Total score is positively correlated with the severity of emotional dysregulation. The BIS‐11‐CV included 30 items under three dimensions: non‐planning (10 items), motor (10 items), and attentional impulsiveness (10 items). Each item is scored on a five‐point Likert scale ranging from 1 (“none”) to 5 (“always”); items within the non‐planning and attentional impulsivity subscales were reverse‐coded. The total score ranges from 30 to 150, with a higher score indicating a higher level of impulsiveness. A reduction of at least 50% in the HDRS‐17 score was considered response and remission was defined as a HDRS‐17 score ≤ 7.

Cognitive function was assessed using the MATRICS Consensus Cognitive Battery (MCCB) [29, 30] including five items: the Trail Making Test (TMT) Part A, the Category Fluency test (Animal Naming), the Spatial Span subtest from the Wechsler Memory Scale‐III (WMS‐III); the Hopkins Verbal Learning Test‐Revised (HVLT‐R), and the Brief Visuospatial Memory Test‐Revised (BVMT‐R). These items were categorized into four cognitive domains according to the MCCB guidelines: processing speed, verbal learning, working memory, and visual learning. Raw scores were converted to demographic‐adjusted scaled scores. Autobiographical memory (AM) was assessed according to the classic Autobiographical Memory Test (AMT) paradigm by Williams and Broadbent [31]. AM induction involved twelve emotional cues, evenly split to negative cues (pain, horror, sadness, loneliness, anger, guilt) and positive cues (proud, successful, honest, happy, fun, brave). Upon presentation of each cue, participants were required to narrate a specific event lasting no longer than 24 h, with a response time limit of 1 min. Recalled events were classified to the following categories: specific memory (SM), extended memory (EM), category memory (CM), non‐memory/omission. EM and CM were collectively referred to as the overgeneral autobiographical memory (OGM), which is one of the most meaningful aspects of AM and considered to be related to depression and suicide [32]. The numbers of cues for which the participants reacted to were used as indicators, and the reaction time between the card presentation and the subject talking about the cue was recorded.

All assessments were completed at baseline and within 48 h after the final MST session.

### 2.3 MST Procedure

The MST treatments were administered under general anesthesia using a Magnetic Therapy stimulator (NS7000, Wuhan China) with a twin‐conical coil (YRD304S). The center of twin coils was positioned at the Cz point (located according to the international standard 10–20 EEG electrode system [33]). Maximum device intensity was set to 100%, with a frequency of 100 Hz and remained stable during the whole treatment. Individual seizure threshold was determined using a titration method, starting at 4 s and incrementing by 2 s until limb convulsions occurred or distinct seizure‐phase waves were recorded on the EEG, up to a maximum of 10 s. Given the established effectiveness and safety of aMST [22], which was also observed in our pilot study, MST sessions were administered once‐daily on consecutive working days until the adolescents achieved remission (HDRS‐17 total score of ≤ 7) or until reached a maximum of eight sessions.

### 2.4 Medication and Anesthesia

Concomitant antidepressant dosages were maintained at a stable level throughout the MST course. Participants undergoing aMST must stop taking benzodiazepines and mood stabilizer (e.g., lithium), the dose, duration of use are presented in the Supporting Information (Table S1). MST was administered under general anesthesia using intravenous propofol (1.5–2.0 mg/kg). Intravenous succinylcholine (1–1.5 mg/kg) was used for muscle relaxation and atropine (0.5–1 mg) was used to reduce airway secretions. Participants were oxygenated until spontaneous respirations returned.

### 2.5 MRI Acquisition and Processing

The structural whole‐brain MRI scans were obtained in 48 h before the first MST session and within 48 h following the last MST session. The scans were performed using a 3.0 T GE scanner at Nanjing Brain Hospital with the following T1‐weighted sequence acquisition parameters: repetition time (TR) = 7.2 ms; echo time (TE) = 2.9 ms; inversion time = 450 ms; flip angle (FA) = 8°; field of view (FOV) = 240 mm × 240 mm; matrix size = 240 × 240; slice thickness = 1 mm, gap = 0 mm; Diffusion tensor imaging (DTI) parameters were as follows: *b*‐value = 1, TR = 9000 ms, TE = 89.1 ms, FOV = 256 mm × 256 mm, matrix = 128 × 128, slice thickness = 2 mm, gap = 0 mm.

### 2.6 Structural MRI Processing

**FIGURE 1:**
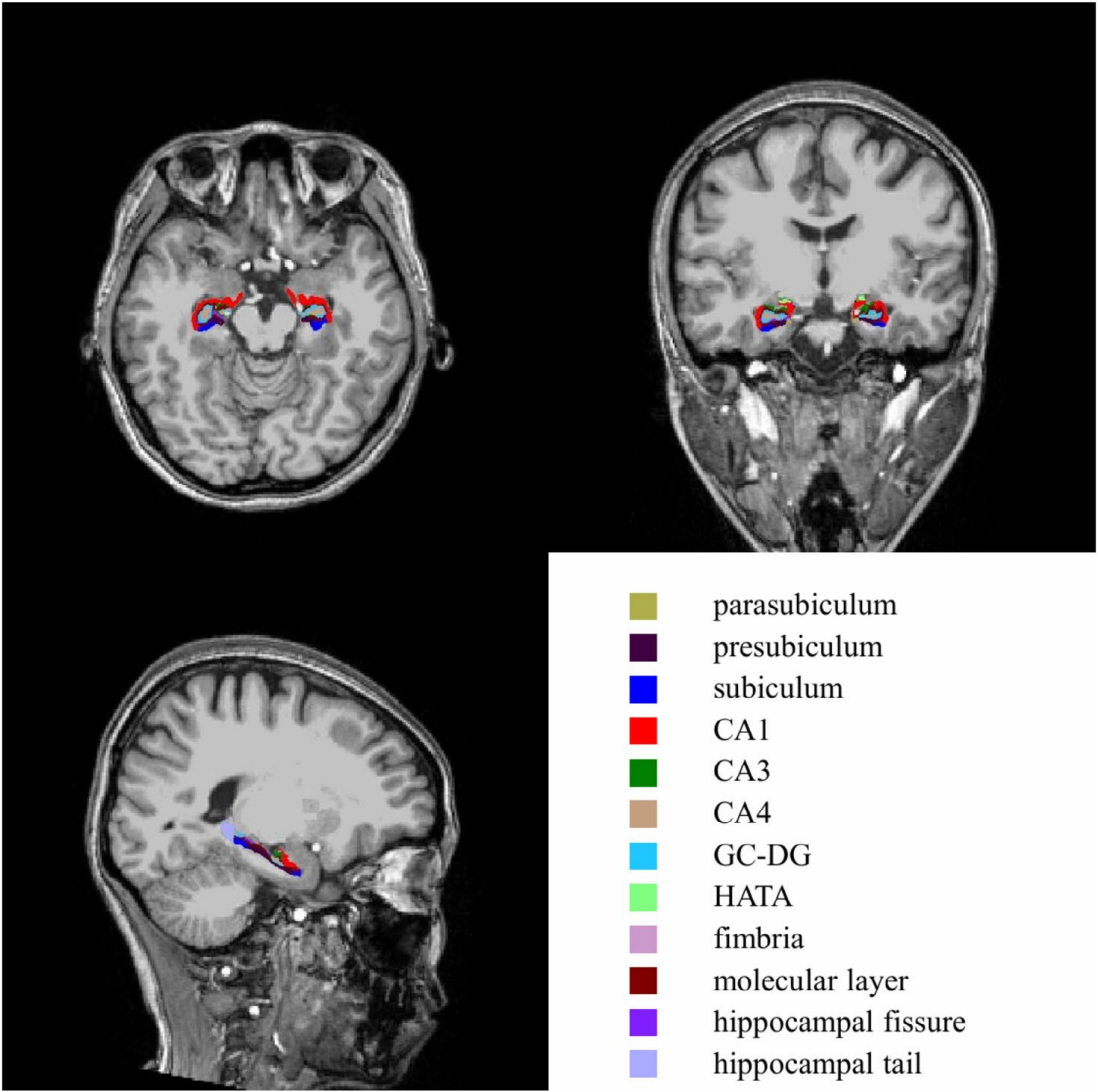
Hippocampal subfield segmentation based on FreeSurfer (v7.1.1). CA, cornu ammonis; GC‐DG, granule cell of the dentate gyrus; HATA, hippocampus‐amygdala transition area.

To determine changes in hippocampal volume, T1‐weighted images were processed using FreeSurfer (v7.1.1). Subcortical structures were automatically segmented via the longitudinal stream to ensure intra‐subject consistency. This pipeline included motion correction, intensity normalization, and reliable atlas registration specifically optimized for longitudinal data (Figure 1).

### 2.7 Diffusion Tensor Imaging (DTI) Processing

Raw DICOM files were converted to NIfTI format using dcm2niix (via MRIcroGL). DTI data were processed using a combination of MRtrix3 (v3.0.4) and FSL (v6.0.7). Initial preprocessing steps included MP‐PCA denoising, Gibbs ringing artifact removal, and B1 field inhomogeneity correction (N4 bias correction) to improve the signal‐to‐noise ratio. Head motion and eddy current‐induced distortions were corrected using the dwifslpreproc command. Following skull stripping using the Brain Extraction Tool (BET), diffusion tensors were estimated using FSL's dtifit. This generated maps for fractional anisotropy (FA), mean diffusivity (MD), axial diffusivity (AD), and radial diffusivity (RD). The DTI metrics were co‐registered to each subject's T1‐weighted image. The T1 images were then normalized to the MNI152 standard space using linear (FLIRT) and non‐linear (FNIRT) registration. By applying the inverse transformation matrix, the JHU ICBM‐DTI‐81 White‐Matter Atlas was warped into the native diffusion space. Finally, mean DTI metrics were extracted from specific regions of interest (ROIs) for statistical analysis. ROIs included the bilateral cingulate gyrus, corpus callosum, hippocampus, uncinate fasciculus, and superior longitudinal fasciculus. Seventeen participants finished the MRI scans before and after aMST.

### 2.8 Statistical Analysis

Statistical analyses were conducted using IBM SPSS Statistics (version 26.0). Baseline and post‐intervention scores for the HDRS‐17, HARS, DERS‐16‐CV, and BIS‐11‐CV, as well as cognitive performance, hippocampal subregion volumes, and white matter microstructural value changes were compared using paired‐sample *t*‐tests. Statistical significance was set at a two‐tailed *p* < 0.05. Effect size was calculated using Cohen's *d*. To control for multiple comparisons across the 12 hippocampal subfields, a Bonferroni correction was applied.

## 3 Result

### 3.1 Demographic and Clinical Characteristics

A total of thirty‐two adolescents diagnosed as bipolar depression finished the aMST treatments and clinical assessments; their ages ranged from 14 to 18 years. Most of the participants were female. All participants were taking antidepressants 1 week before the first MST session. To facilitate statistical analysis, all antidepressant dosages were converted into fluoxetine equivalents. Overall, thirty‐two participants received an average of six aMST sessions, with seizure duration around 47 s (Table 1).

**TABLE 1:** Demographic and clinical characteristics.

Population	Value
Sample size, *n*	32
Sex, *n* (%)	
Male	7 (21.9%)
Female	25 (78.1%)
Age, mean ± SD	16.06 ± 1.70
Illness duration, mean ± SD	1.88 ± 1.62
Family history, *n* (%)	
Yes	12 (37.5%)
No	20 (62.5%)
Age of first NSSI, year, mean ± SD	14.38 ± 1.72
Dosage of Fluoxetine, mg, mean ± SD	24.61 ± 18.35
Number of MST sessions, times, mean ± SD	6.44 ± 1.65
Average seizure duration, second, mean ± SD	47.40 ± 25.43
Average index of seizure energy, μV^2^, mean ± SD	147424.29 ± 185,253
Inhibition index after seizure, %, mean ± SD	97.53 ± 13.46
Clinical response, *n* (%)	20 (62.5%)
Clinical remission, *n* (%)	14 (43.8%)
Completed MRI scans, *n* (%)	17 (53.1%)

*Note:* Clinical response was defined as a ≥ 50% decrease in HDRS‐17 scores. Clinical remission was defined as a HDRS‐17 score ≤ 7.

Abbreviations: MST, magnetic seizure therapy; NSSI, non‐suicidal self‐injury.

### 3.2 Clinical Outcomes

Clinical outcomes were assessed at baseline and within 48 h after aMST treatment. Twenty patients (62.5%) showed clinical response to aMST (a reduction of at least 50% in the HDRS‐17 score) and fourteen patients (43.8%) reached remission (HDRS‐17 score ≤ 7). Following aMST, participants demonstrated significant reductions in both HDRS‐17 score (*t* = 14.250; *p* < 0.001) and HARS score (*t* = 7.124; *p* < 0.001). There was a significant decrease in BIS‐11 total score (*t* = 5.554; *p* < 0.001) and its items including non‐planning impulsiveness (*t* = 3.714; *p* = 0.001), motor impulsiveness (*t* = 6.951; *p* < 0.001), attentional impulsiveness (*t* = 3.790; *p* = 0.001). Significant score decreases were also observed in DERS‐16 (*t* = 6.154; *p* < 0.001) after aMST (Table 2).

**TABLE 2:** Therapeutic effects of aMST on depressive symptoms, anxiety, impulsivity, and emotion regulation.

	Pre‐MST (*n* = 32)	Post‐MST (*n* = 32)	*t*	*p*	Cohen's *d*
BIS‐11 score, mean ± SD					
Non‐planning impulsiveness	49.06 ± 10.37	40.47 ± 15.77	3.714	0.001*	0.66
Motor impulsiveness	61.88 ± 16.26	41.02 ± 15.04	6.951	0.000*	1.23
Attentional impulsiveness	50.63 ± 12.43	39.53 ± 14.06	3.790	0.001*	0.67
Total	53.85 ± 10.86	40.34 ± 13.28	5.554	0.000*	0.98
DERS‐16 score, mean ± SD	51.03 ± 14.89	37.19 ± 12.95	6.154	0.000*	1.09
HDRS‐17 score, mean ± SD	23.52 ± 3.85	9.14 ± 4.67	14.250	0.000*	2.52
HARS score, mean ± SD	21.54 ± 7.17	12.57 ± 4.79	7.124	0.000*	1.26

*Note:* Significant differences were found across all scales. Data was expressed as mean ± SD. *p* values were obtained using paired *t*‐test. Effect size was calculated using Cohen's *d*.

Abbreviations: BIS, Barratt Impulsiveness Scale; DERS, Difficulties in Emotion Regulation Scale; HARS, Hamilton Anxiety Rating Scale; HDRS, Hamilton Depression Rating Scale.

* *p* < 0.05.

Patients showed statistically improvements in the score of most MCCB domains, including speed of processing (*t* = −4735; *p* < 0.001); working memory (*t* = −2.803; *p* = 0.013), visual learning (*t* = −3.256; *p* = 0.005). There were no statistically significant differences in verbal learning scores after aMST (*t* = 0.712; *p* = 0.487), although a downward trend was observed. The scores of OGM for the positive cues after aMST treatment were significantly lower than at baseline (*t* = 3.162; *p* = 0.007). No significant change of the scores of OGM for the negative cues was observed after aMST (*t* = 1.633; *p* = 0.125). Despite the lack of statistical significance, our study observed upward trends in scores of both SM for the positive cues (*t* = −1.284; *p* = 0.220) and SM for the negative cues (*t* = −1.483; *p* = 0.160) following the aMST (Table 3).

**TABLE 3:** Impact of aMST on neurocognitive domains and autobiographical memory.

	Pre‐MST (*n* = 32)	Post‐MST (*n* = 32)	*t*	*p*	Cohen's *d*
MCCB score, mean ± SD					
Speed of processing	50.06 ± 11.66	58.76 ± 12.22	−4.735	0.000*	0.84
Working memory	36.29 ± 9.57	41.82 ± 6.64	−2.803	0.013*	0.50
Verbal learning	47.29 ± 8.27	45.71 ± 9.88	0.712	0.487	0.13
Visual learning	52.94 ± 8.30	58.65 ± 3.92	−3.256	0.005*	0.58
AMT, mean ± SD					
SM for the positive cues	4.00 ± 1.51	4.47 ± 1.41	−1.284	0.220	0.23
SM for the negative cues	3.67 ± 1.45	4.53 ± 1.41	−1.483	0.160	0.26
OGM for the positive cues	1.40 ± 1.21	0.73 ± 0.88	3.162	0.007*	0.56
OGM for the negative cues	1.93 ± 1.34	1.13 ± 1.25	1.633	0.125	0.29

*Note:* There were significant differences found in the speed of processing, working memory, visual learning, OGM for the positive cues. Data was expressed as mean ± SD. *p* values were obtained using paired *t*‐test. Effect size was calculated using Cohen's *d*.

Abbreviations: AMT, autobiographical memory test; MCCB, MATRICS Consensus Cognitive Battery; OGM, overgeneral autobiographical memory; SM, specific memory.

* *p* < 0.05.

### 3.3 Neuroimaging Outcomes

No significant difference was found in the volumes of bilateral hippocampal subregions after aMST such as cornu ammonis 1 (CA1), CA3, CA4, Granule Cell of the Dentate Gyrus (GC‐DG), subiculum, presubiculum, parasubiculum, molecular layer, hippocampal tail, fimbria, hippocampal fissure and hippocampus‐amygdala transition area (HATA) (Table 4).

**TABLE 4:** Volumetric alterations of bilateral hippocampal subfields before and after aMST.

Hippocampal subfield, mm^3^	Pre‐MST (*n* = 17)	Post‐MST (*n* = 17)	*t*	*p*	Cohen's *d*
Left					
CA1	541.94 ± 44.78	540.89 ± 49.27	0.253	0.804	0.06
CA3	174.61 ± 23.72	174.52 ± 25.46	0.035	0.973	0.01
CA4	281.28 ± 30.85	235.37 ± 23.88	0.671	0.513	0.16
GC‐DG	276.6 ± 24.64	274.71 ± 22.63	0.738	0.472	0.18
Subiculum	378.48 ± 40.96	374.19 ± 35.83	1.013	0.327	0.25
Presubiculum	281.28 ± 30.85	278.79 ± 29.42	0.605	0.554	0.15
Parasubiculum	61.25 ± 11.38	59.23 ± 11.71	2.307	0.069	0.56
Molecular layer	522.39 ± 36.51	520.15 ± 34.21	0.618	0.546	0.15
Hippocampal tail	555.88 ± 68.65	552.07 ± 57.37	0.956	0.354	0.23
Fimbria	67.68 ± 14.64	67.15 ± 10.73	0.234	0.818	0.06
Hippocampal fissure	197.17 ± 30.96	195.31 ± 18.7	0.388	0.703	0.09
HATA	38.94 ± 5.06	47.21 ± 32.86	−1.094	0.291	0.27
Whole hippocampus	3135.98 ± 239.19	3116.4 ± 213.79	0.954	0.355	0.23
Right					
CA1	578.64 ± 63.67	571.9 ± 59.84	1.535	0.146	0.37
CA3	192.15 ± 21.38	192.8 ± 19.59	−0.289	0.777	0.07
CA4	257.44 ± 24.82	251.37 ± 22.55	0.577	0.573	0.14
GC‐DG	289.27 ± 25.07	287.45 ± 23.27	0.605	0.554	0.15
Subiculum	378.5 ± 37.07	374.17 ± 32.89	1.111	0.284	0.27
Presubiculum	257.44 ± 24.82	255.71 ± 23.91	0.519	0.612	0.13
Parasubiculum	61.71 ± 11.31	59.16 ± 7.38	1.790	0.094	0.43
Molecular layer	543.75 ± 42.55	539.99 ± 42.22	0.817	0.427	0.20
Hippocampal tail	562.65 ± 66.86	552.61 ± 67.59	2.133	0.050	0.52
Fimbria	65.07 ± 12.2	64.68 ± 11.74	0.220	0.829	0.05
Hippocampal fissure	195.38 ± 29.72	199.84 ± 30.54	−1.164	0.263	0.28
HATA	39.81 ± 5.13	39.66 ± 5.79	0.218	0.830	0.05
Whole hippocampus	3221.94 ± 248.85	3189.5 ± 236.97	1.265	0.225	0.31

*Note:* No significant difference was found in the volumes of bilateral hippocampal subregions before and after aMST. Data was expressed as mean ± standard deviation SD. *p* values were obtained using a paired *t*‐test. Effect size was calculated using Cohen's *d*.

Abbreviations: CA, cornu ammonis; GC‐DG, granule cell of the dentate gyrus; HATA, hippocampus‐amygdala transition area.

**FIGURE 2:**
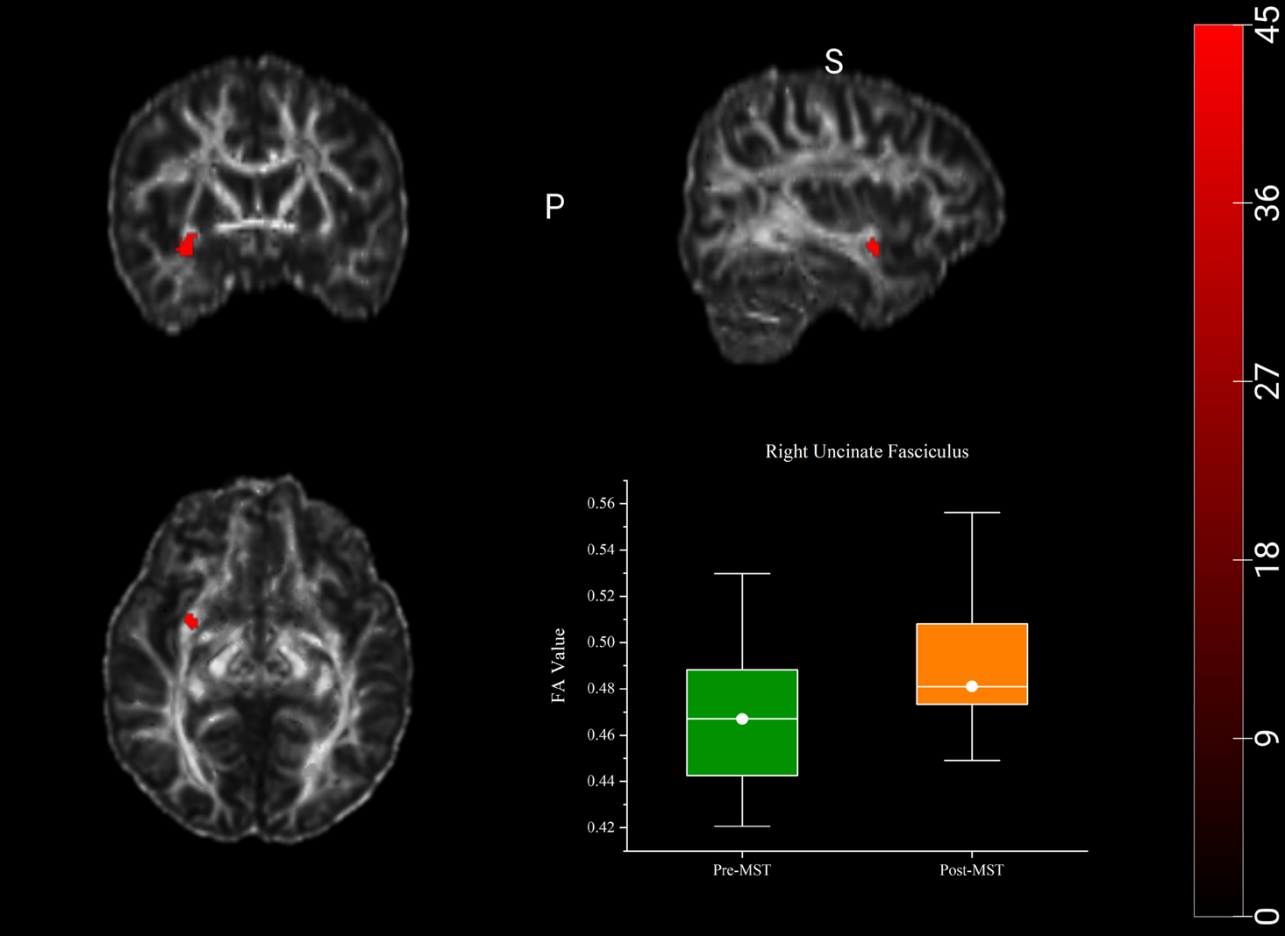
Changes in diffusion metrics within the right uncinate after aMST. There were increased FA values in the right uncinate fasciculus (*p* < 0.05). Data is presented in the form of box plots. *p* values were obtained using paired *t*‐test. The highlighted red areas represent the right uncinate fasciculus. FA, fractional anisotropy.

**FIGURE 3:**
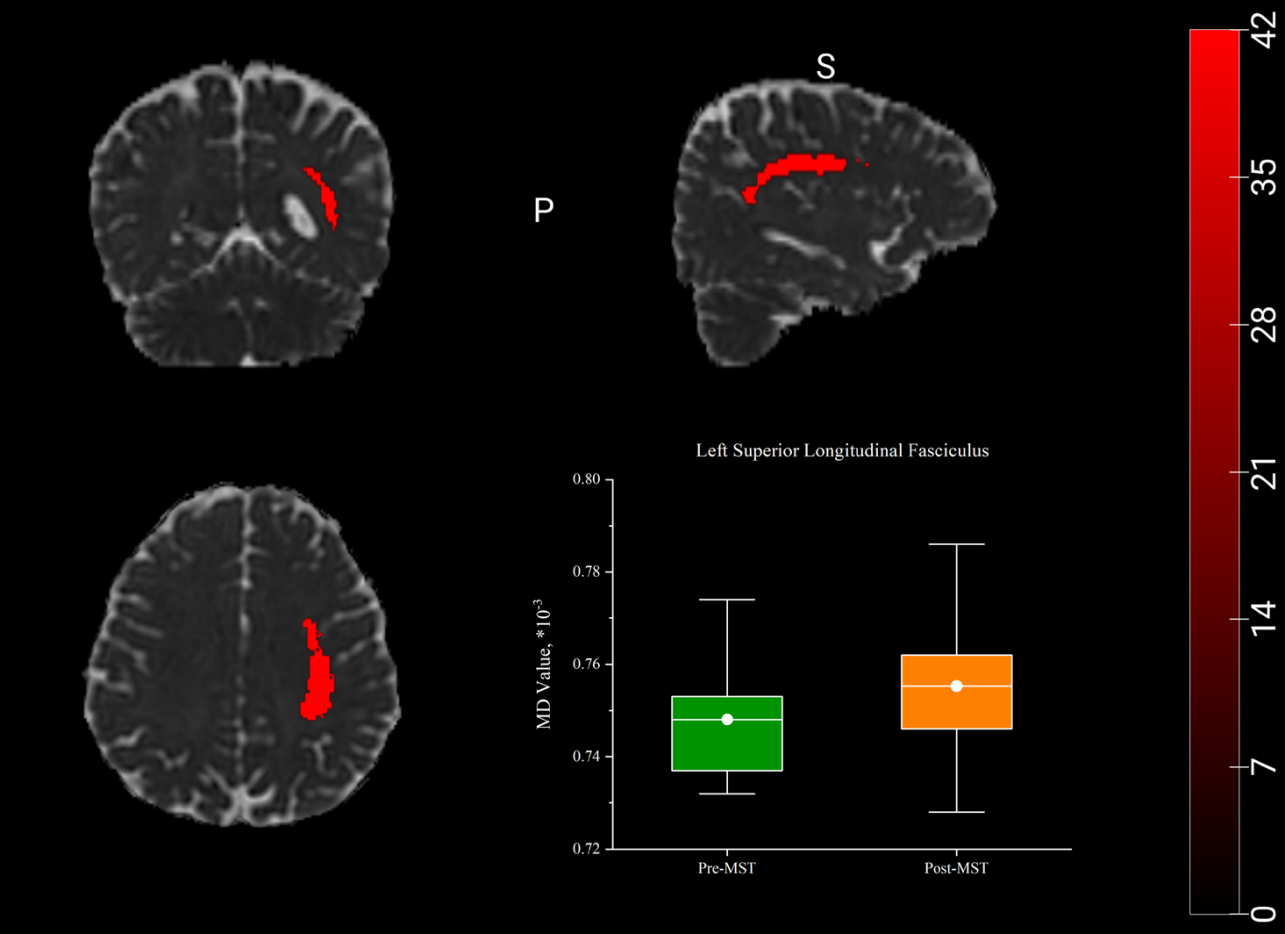
Changes in diffusion metrics within the left superior longitudinal fasciculus after aMST. There were increased MD values in the left superior longitudinal fasciculus (*p* < 0.05). Data is presented in the form of box plots. *p* values were obtained using paired *t*‐test. The highlighted red areas represent the left superior longitudinal fasciculus. MD, mean diffusivity.

Analyses of DTI metrics were conducted for the regions of interest (ROIs), including bilateral cingulate gyrus, corpus callosum, hippocampus, uncinate fasciculus, and superior longitudinal fasciculus. The results showed an increase in FA within the right uncinate fasciculus (*t* = −2.238; *p* = 0.040) (Figure 2) and an increase in MD (*t* = −2.427; *p* = 0.027) within the left superior longitudinal fasciculus (Figure 3). In other ROIs, no significant changes were observed in any DTI‐related metrics.

## 4 Discussion

The aim of the present study was to investigate the effects of aMST on clinical symptoms, cognitive functions, hippocampal subfield volumes and white matter microstructure in adolescents with bipolar depression and comorbid NSSI. The results showed aMST was associated with significant improvements in depressive and anxiety symptoms, accompanied by reductions in impulsivity and difficulties in emotion regulation. Moreover, adolescents with bipolar depression and NSSI did not show the global cognitive deterioration following treatment. Multiple cognitive domains including speed of processing, working memory and visual learning showed improvement compared with baseline levels. Autobiographical memory test revealed a selective reduction in OGM for the positive cues, while no significant changes were observed for the negative cues. Notably, while hippocampal subfield volumes remained stable post‐treatment, neuroimaging data revealed specific changes in white matter microstructural integrity, which included an increase in fractional anisotropy (FA) in the right uncinate fasciculus, alongside elevated mean diffusivity (MD) in the left superior longitudinal fasciculus. These findings suggest that aMST was associated with significant clinical improvement in adolescents with comorbid bipolar depression and NSSI, also enhanced cognitive performance in several domains. The alterations in white matter microstructure may represent one of the potential neurobiological correlates underlying the observed clinical improvements.

In our previous study, we did not find cognitive impairments after consecutive sessions of MST. Furthermore, accelerating the frequency of MST (five times once a week) is related to the better prognosis in adolescents with comorbid bipolar depression and NSSI. This method has been confirmed by previous research, which indicated aMST was well tolerated without significant evidence of cognitive side effects and produced rapid improvement in depression symptoms [22]. Thirty‐two patients meeting the inclusion criteria completed the whole procedure and twenty patients received clinical response after at least four MST sessions (response rate 62.50%). The observed clinical improvements following aMST are broadly consistent with prior studies reporting robust antidepressant effects of MST [19, 34, 35]. Following MST, the study showed that the scores of the 24‐item Hamilton Depression Rating Scale (HDRS‐24) in patients with bipolar depression decreased significantly, accompanied by a reduction in suicidal ideation and 44.7% of participants achieving complete remission (a total score of Beck Scale for Suicidal Ideation = 0) [18]. For adolescents, a case report by Blumberger et al. [36] provided initial evidence supporting the effectiveness of MST in adolescents with bipolar depression. Later research further supported the finding that MST is associated with clinical efficacy similar to ECT when treating adolescents with depression [20, 21]. The present study suggests that aMST is effective for adolescents with comorbid bipolar depression and NSSI.

Beyond symptomatic improvement, the present findings also showed changes in core psychological processes that are highly relevant to NSSI [37, 38], such as the score decline on BIS‐11 (no planning, motor and attentional impulsiveness) and DERS‐16 at the end of aMST. Prior randomized controlled trials in high‐suicide‐risk adolescents have demonstrated that improvements in emotion regulation, which were exhibited by reductions in DERS scores, mediate subsequent decreases in self‐harming and suicidal behaviors [39]. Complementary evidence from neuromodulation studies further suggested that interventions targeting prefrontal control circuits such as transcranial direct current stimulation (tDCS) and repetitive transcranial magnetic stimulation (rTMS) can reduce impulsivity, reflected by lower BIS scores, thereby reducing self‐harm behavior [40]. Within this, the therapeutic effects of MST in adolescents with comorbid bipolar disorder and NSSI may be partially explained by concurrent reductions in impulsivity and enhancements in emotion regulation capacity, which strengthen the control over affective and behavioral responses and ultimately reduce self‐harm risk. However, it remains unclear whether these changes represent direct effects of aMST or are partially mediated by overall improvements in depressive symptoms. Nevertheless, our research suggested adolescents with comorbid bipolar depression and NSSI could benefit from MST, whose self‐harm behaviors were alleviated within 2 weeks and depression symptoms moderated as well.

The present study did not find severe cognitive impairment after aMST in adolescents with comorbid bipolar depression and NSSI. On the contrary, there were significant improvements in several cognitive fields including speed of processing, working memory, and visual learning. These results were broadly consistent with previous reports suggesting that MST may account for less cognitive deterioration in domains such as visuospatial, language, attention and executive function to a greater extent than traditional electroconvulsive therapy (ECT) [34, 41]. However, verbal learning, which relies more heavily on encoding and consolidation processes [42], remained largely unchanged, which has also been reported in a previous study [43]. While no statistically significant differences were detected, verbal learning scores exhibited a mild downward trend. During adolescence, cognitive systems are characterized by heightened plasticity following intensive interventions [44], at the same time, this increased plasticity may render certain cognitive functions more sensitive to short‐term alterations during intensive treatment. Taken together, the overall cognitive improvement indicates that aMST may be more tolerable and effective in adolescents.

Autobiographical memory (AM) is commonly used as an index of retrograde memory, as it reflects the retrieval of personally experienced events acquired prior to treatment. Many studies have indicated that ECT has cognitive side effects, especially in AM, while MST was not associated with severe AM impairment [45, 46]. In our study, overall AM performance remained largely stable following aMST, with a reduction in the score of OGM for the positive cues being observed. Increased availability of negative categoric memories and the attenuation of positive specific recall represent vulnerabilities for patients with depression [47]. The observed reduction may reflect transient modulation of affective memory processing during a period of rapid clinical improvement, which may be related to depressive remission. Importantly, this pattern differs from the diffuse autobiographical memory disturbances which are often reported following ECT and further supports the cognitive safety of aMST in adolescents with comorbid bipolar depression and NSSI.

The hippocampus is a critical structure of the central nervous system, playing essential roles in cognition (learning, memory consolidation, and emotional regulation) [48]. The hippocampus comprises multiple anatomically and functionally distinct subfields (e.g., CA1, CA3, dentate gyrus, and subiculum), which may show differential sensitivity to psychiatric disorders and neuromodulation therapies [49]. In the present study, we found no significant alteration in total bilateral hippocampal volume and hippocampal subfield volumes of adolescents with comorbid bipolar depression and NSSI following the completion of MST. Previous research reported robust volumetric increases across multiple hippocampal subregions following ECT, whereas no significant subfield changes were observed after MST in patients with schizophrenia [50]. Consistent with these findings, aMST was not associated with volumetric alterations in hippocampal subfields. MST uses high‐frequency magnetic fields that penetrate the skull with minimal loss, producing seizure induction with substantially more focal cortical stimulation compared with the diffuse electrical fields of ECT [51]. As magnetic pulses are relatively confined to the superficial cortex and do not penetrate deeply into subcortical areas such as the hippocampus, MST may achieve clinical symptom relief while sparing deeper structures implicated in memory and cognition, which may contribute to cognitive protection. Taken together, these results suggest that, in contrast to ECT, aMST may achieve clinical efficacy without inducing pronounced structural remodeling of the hippocampus, a region closely implicated in the cognitive side effects of convulsive therapies.

To further investigate treatment‐related microstructural alterations beyond gray matter morphology, DTI was employed to characterize white matter integrity following aMST. We observed microstructural alterations in several white matter tracts (right uncinate fasciculus and left superior longitudinal fasciculus) following aMST, primarily characterized by changes in diffusion metrics such as fractional anisotropy (FA) and mean diffusivity (MD). FA is commonly interpreted as an index of fiber coherence, axonal organization, and myelination [52]. The uncinate fasciculus connects the orbitofrontal cortex with anterior temporal and limbic regions, including the amygdala and hippocampus, and plays a critical role in emotional regulation, impulse control, and fronto‐limbic integration [53]. Increased FA in the right uncinate fasciculus following aMST could therefore reflect enhanced microstructural organization within emotion‐regulatory pathways, which is particularly relevant in adolescents with bipolar disorder and NSSI. However, this does not prove that MST reduces NSSI behavior by acting on the emotion‐regulation pathway; these changes could represent a neuroplastic effect of aMST on white matter microstructure, possibly related to repeated seizure induction and neuromodulation. And it could also be secondary to symptomatic improvement, reflecting state‐dependent alterations rather than direct treatment effects. Alternatively, these findings could even be unrelated to either treatment or clinical response, representing incidental observations or methodological artifacts. Mean diffusivity (MD) reflects the overall magnitude of water diffusion and is sensitive to changes in tissue density and microstructural complexity [54]. The superior longitudinal fasciculus is a major fronto‐parietal association tract involved in executive function, attention, and language‐related processing [55, 56]. The observed increase in MD in the left superior longitudinal fasciculus may indicate transient microstructural reorganization. Considering the absence of significant hippocampal volumetric changes and the trend toward reduced verbal learning performance, these findings suggest that MST may exert domain‐specific effects on neural circuits. But whether increased MD represents beneficial reorganization, temporary disruption, or a nonspecific effect remains unclear. The observed transient change cannot be confirmed without longitudinal imaging beyond the treatment period. Overall, the combined gray matter, white matter, and cognitive findings may support a model in which aMST induces selective and developmentally mediated neuroplastic changes.

### 4.1 Limitations

Several limitations of this pilot study must be acknowledged. First, the present study was a single‐arm, open‐label trial without a control group, which to some extent weakens the persuasiveness of the actual effect of aMST, because the placebo effect, the synergistic effect of drug treatment, and the influence of disease progression cannot be completely ruled out. Future randomized controlled trials (RCTs), such as an ECT group, a sham MST condition, or a standard pharmacotherapy, are necessary to further establish the effect of aMST. Second, despite the study being designed as a pilot investigation to explore feasibility, safety, and preliminary mechanism of aMST, the small sample size may increase the risk of Type II errors and limit the detection of subtle neurobiological effects. The majority of our sample consists of female teenagers. This proportion is in line with the gender distribution of NSSI adolescents, but it is necessary to increase the sample size and balance the gender ratio in order to avoid the influence of confounding variables. Although the medication strategy was kept stable during the intervention period, the potential contribution of concomitant pharmacotherapy to the overall therapeutic effects cannot be completely ignored. Finally, this study only investigated the acute effects of aMST; the long‐term follow‐up imaging and cognitive assessments are still underway.

## 5 Conclusion

In conclusion, this study provides clinical, cognitive, and neuroimaging evidence supporting the efficacy and neurocognitive safety of aMST in adolescents with comorbid bipolar depression and NSSI. aMST was associated with meaningful clinical improvement and ameliorative cognitive performance across multiple domains. Neuroimaging data further demonstrated stable hippocampal subfield volumes and selective white matter microstructural alterations within fronto‐limbic and fronto‐parietal pathways. Together, these results indicate that aMST may offer an effective convulsive neuromodulation approach with a favorable cognitive profile for adolescents with comorbid bipolar depression and NSSI. Future studies with larger samples, longer follow‐up, and multimodal imaging will be essential to clarify the durability and clinical significance of these neurobiological changes.

## Author Contributions

Ruiqiu Zhu, Zhuma Jin, and Yuxiu Sui conceived and designed the study. Ruiqiu Zhu, Peiyu Cao, Yilin Tang, Yucheng Lin, Yifan Wang, Yantong Li, and Ran Tao performed data acquisition, analysis, and interpretation. All authors contributed to drafting and critically revising the manuscript for important intellectual content. All authors read and approved the final version of the manuscript.

## Ethics Statement

The study was approved by the Ethics Committee of the Affiliated Brain Hospital of Nanjing Medical University (2025‐KY075‐01).

## Consent

Written informed consent was obtained from all patients and their legal guardians.

## Conflicts of Interest

The authors declare no conflicts of interest.

## Supporting information


**Table S1:** The medications prior to aMST.

## Data Availability

The data that support the findings of this study are available on request from the corresponding author. The data are not publicly available due to privacy or ethical restrictions.
