# Supplementary material for: Accelerated Magnetic Seizure Therapy for Non‐Suicidal Self‐Injury in Adolescents With Bipolar Depression
Source: CNS Neurosci Ther. 2026 Apr 21;32(4):e70880. doi: 10.1002/cns.70880 (PMC13098537; doi:10.1002/cns.70880)
Supplement: Supplementary file 1 — Table S1: The medications prior to aMST. [file CNS-32-e70880-s001.docx]

|  | Number of Users (n) | Dose (mg) | Duration of Use (days) |
| --- | --- | --- | --- |
| Lithium | 12 | 550.00±116.78 | 3.75±1.55 |
| Lamotrigine | 2 | 31.25±26.52 | 3.50±2.12 |
| Lorazepam | 26 | 1.08±0.42 | 5.12±2.93 |

**Supplementary Table.1 The medications prior to aMST.**

The medications prior to aMST including lithium, lamotrigine and lorazepam. All medications were withheld at least 24 hours prior to the first aMST. Data was expressed as mean ± SD.
